# Trends in cardiometabolic multimorbidity in non-elderly adult Medicaid enrollees, 2018–2022

**DOI:** 10.3389/fepid.2025.1571650

**Published:** 2025-06-26

**Authors:** Puneet Kaur Chehal, Pooja Dilip Lalwani, Erin C. Fuse Brown, Mohammed K. Ali, Solveig A. Cunningham

**Affiliations:** ^1^ Department of Family and Preventative Medicine, Emory School of Medicine, Atlanta, GA, United States; ^2^ Department of Biology, Duke University, Durham, NC, United States; ^3^ Department of Health Services, Policy & Practice, Brown University School of Public Health, Providence, RI, United States; ^4^ Emory Global Diabetes Research Center, Woodruff Health Sciences Center, Emory University, Atlanta, GA, United States; ^5^ Hubert Department of Global Health, Emory University, Atlanta, GA, United States

**Keywords:** hypertension, hyperlipidemia, cardiovascular diseases, Medicaid, multimorbidity, diabetes, obesity

## Abstract

**Importance:**

Medicaid, as the largest U.S. insurer, can reduce cardiometabolic multimorbidity.

**Objective:**

Assess patterns and trends in cardiometabolic multimorbidity among Medicaid-enrolled adults.

**Design:**

Analysis of 2018–2022 National Health Interview Survey data, a nationally representative cross-sectional survey.

**Conditions Studied:**

Hypertension, hyperlipidemia, coronary heart disease, angina, heart attack, stroke, diabetes, and obesity.

**Setting:**

U.S., 2018–2022.

**Participants:**

11,090 adults (19–64 years) with Medicaid coverage.

**Main Outcomes:**

Proportion with one or multiple cardiometabolic conditions.

**Findings:**

(a) 29.3% had one cardiometabolic condition; 29.7% had multimorbidity: 14.5% with 2, 8.0% with 3, and 7.1% with 4+ conditions. (b) Obesity, hypertension, and hyperlipidemia were the most common conditions either individually or together. (c) Obesity was more common in women than men, and women were more likely to have a single condition while men were more likely to have multimorbidity; these differences between men and women were larger in younger adults (<41 years) than older adults. (d) There was higher multimorbidity among older, non-working, and less educated Medicaid enrollees. (e) Prevalence of multimorbidity over time did not change but there was a decrease in the proportion of enrollees with no conditions which was offset by an increase in enrollees with a single condition.

**Conclusion:**

29.7% of Medicaid-insured adults had cardiometabolic multimorbidity, and another 29.3% were at risk for it. Potential cuts to Medicaid coverage may exacerbate the burden of cardiometabolic multimorbidity in Medicaid enrollees.

## Introduction

In the US, state Medicaid programs provide subsidized public health insurance for low-income populations who are more likely to develop chronic cardiovascular and metabolic conditions relative to the general population (1). Cardiometabolic multimorbidity (diagnosis of 2 or more cardiovascular or metabolic chronic conditions, e.g., diabetes and heart disease) increases with age (2, 3). When poorly managed, individual cardiometabolic conditions also increase the risk for additional disease and costly disabling complications (e.g., amputation from diabetes). Research-backed preventive care and disease management strategies exist for cardiometabolic conditions but disease management can be challenging for low-income or uninsured populations (4–6).

It is unclear how cardiometabolic multimorbidity manifests in contemporary adult Medicaid enrollees, which include working-age adults made eligible for Medicaid by the Affordable Care Act (ACA). Published reports on multimorbidity among Medicaid enrollees used pre-ACA data from the early 2000s and excluded majority-managed-care states altogether (7, 8). Pre-ACA, otherwise non-disabled, low-income working-age adults with chronic conditions would typically only qualify for Medicaid when and if their conditions progressed enough to cause a qualifying disability. Post-ACA, all income eligible adults have access to Medicaid in 40 of 50 states (and in the District of Columbia) largely through managed care programs (9). By understanding the epidemiology of cardiometabolic multimorbidity in newer non-elderly adult Medicaid-insured populations, policymakers can introduce timely interventions that target enrollees at risk of worsening cardiometabolic multimorbidity.

Our research contributes a national, post-ACA portrait of cardiometabolic multimorbidity prevalence and trends among non-elderly Medicaid- insured adults (2018–2022). We additionally stratify our sample to compare prevalence of cardiometabolic conditions and combinations of conditions across age and sex.

## Methods

We used data from the National Health Interview Survey (NHIS), a nationally representative annual cross-sectional survey conducted by the National Center for Health Statistics (NCHS) to monitor the health of the US civilian non-institutionalized population (10). We examined data for all adults 19–64 years of age with Medicaid coverage. Data collection protocols for the NHIS are reviewed and approved by the NCHS Ethics Review Board. This study was exempt from institutional review board review because the data used were publicly available data with no identifying patient health information. The study followed the Strengthening of the Reporting of Observational Studies in Epidemiology reporting guidelines (21). Our final analytical sample includes information from 11,090 respondents.

We used self-reported information on medical conditions collected from an adult randomly selected in each family. For the purposes of this study, we considered the following cardiometabolic conditions: hypertension, hyperlipidemia (cholesterol), coronary heart disease, angina pectoris, heart attack, stroke, diabetes, and obesity. Other important conditions such as heart failure, chronic kidney disease (CKD), insulin resistance, and non-alcoholic fatty liver disease were not observable in the data. Sample adults are asked whether a medical professional had told them they had a given condition except for obesity which is calculated by NHIS based on self-reported height/weight information (10). Cardiometabolic multimorbidity is defined as having 2 or more cardiometabolic conditions as indicated in the data. Medicaid coverage was identified using survey questions on whether sample adults had any health insurance coverage and the type of health insurance coverage if applicable.

To characterize common cardiometabolic disease patterns, we report prevalence of 1 condition, 2 conditions, 3 conditions, and 4 or more conditions. We report prevalence across years by number of conditions and the cumulative density function (CDF) of total cardiometabolic conditions. To explore differences in prevalence of any cardiometabolic condition and multimorbidity across sociodemographic groups, we considered sex (female or male), age (19–24, 25–29, 30–34, 35–39, 40–44, 45–49, 50–54, 55–59, 60–64), race/ethnicity (non-Hispanic White, non-Hispanic Black, Hispanic, and other), education (high school or less, some college, or 4 years or more of higher education), employment status (employed, retired or not working), and marital status (married or living with a partner or not married). Our analysis of specific conditions and condition combinations report estimates by sex or binary age category (<41, ≥41, where 41 is the median age of the Medicaid enrollees in the sample.

The Sample Adult Weight included in the NHIS data was used to account for the complex survey design, non-response and post-stratification adjustment. We report weighted nationally representative estimates except if noted otherwise, and 95% confidence intervals (CI) with estimates.

## Results

Table 1 shows the prevalence of 0, 1, 2, 3, and 4 or more cardiometabolic conditions for the overall and subpopulations of Medicaid enrollees. 41.0% of enrollees had no conditions while 29.3%, 14.5%, 8.0%, and 7.1% of enrollees had 1, 2, 3, or 4 or more conditions respectively. Collectively, 29.6% of Medicaid enrollees had cardiometabolic multimorbidity of any level (Table 1). More women had 1 cardiometabolic condition (25.5% for men, 31.8% for women) but more men had cardiometabolic multimorbidity with three conditions or four or more conditions (8.7% and 8.5% for men, and 7.6% and 6.2% for women). More non-Hispanic Black and White enrollees had 2, 3, 4 or more diagnoses than Hispanic adults, e.g., prevalence of 2 diagnoses for Black, White and Hispanic enrollees were 16.5%, 14.8% and 13.2% respectively. Larger proportions of adults with a high school degree or less had multiple conditions relative to adults with some higher education (e.g., among adults with 3 diagnoses, 9.0%, 6.8% and 6.1% had a high school degree or less, some college, and four years of college or more respectively). 32% of employed adults had one diagnosis compared to 27%–27.4% of retired or unemployed adults, whereas 2, 3, 4 or more diagnoses were more common in retired or unemployed adults.

**Table 1:** Proportion of cardiometabolic conditions among Medicaid enrollees, 2018–2022.

Characteristics	Total Medicaid enrollees, *n*	0 condition	1 condition	Dyad	Triad	4 or more
		% (95% CL)	% (95% CL)	% (95% CL)	% (95% CL)	% (95% CL)
Overall	11,090	41.0 (39.6–42.4)	29.3 (28.3–30.3)	14.5 (13.6–15.5)	8.0 (7.5–8.6)	7.1 (6.5–7.8)
Sex						
Male	3,994	43.5 (41.4–45.5)	25.5 (23.8–27.2)	13.9 (12.7–15.2)	8.7 (7.7–9.8)	8.5 (7.5–9.6)
Female	7,096	39.4 (37.8–40.9)	31.8 (30.5–33.2)	15.0 (14.0–16.0)	7.6 (6.9–8.3)	6.2 (5.5–7.1)
Age						
19–24	1,214	65.0 (61.8–68.0)	27.0 (24.3–30.0)	6.3 (4.7–8.2)	1.3 (0.6–2.9)	0.4 (0.1–1.2)
25–29	1,274	52.5 (48.7–56.2)	36.4 (32.6–40.5)	8.5 (7.0–10.3)	2.3 (1.3–3.9)	0.3 (0.1–0.9)
30–34	1,447	48.9 (44.9–53.0)	35.0 (31.7–38.5)	11.7 (10.0–13.6)	3.1 (2.2–4.5)	1.3 (0.8–2.0)
35–39	1,260	41.5 (38.3–44.7)	34.0 (30.9–37.3)	15.7 (13.4–18.2)	5 (4.0–6.3)	3.8 (2.6–5.6)
40–44	1,128	36.9 (33.9–40.0)	30.9 (27.5–34.5)	17.7 (15.3–20.5)	9.8 (7.8–12.4)	4.7 (3.5–5.6)
45–49	935	29.7 (26.3–33.4)	28.2 (25.0–31.6)	18.5 (15.7–21.6)	12.5 (10.0–15.5)	11.1 (8.5–14.4)
50–54	1,065	25.6 (22.0–29.5)	23.5 (20.3–27.0)	22.1 (18.9–25.6)	14.7 (12.4–17.4)	14.2 (12.1–16.5)
55–59	1,348	20.7 (18.2–23.6)	19.2 (16.7–21.9)	21.4 (18.1–25.1)	18 (15.5–20.7)	20.8 (17.3–24.6)
60–64	1,419	16.1 (14.1–18.4)	24.1 (21.2–27.2)	21.3 (18.2–24.7)	17.7 (15.3–20.5)	20.8 (18.4–23.5)
Race/ethnicity						
Non-Hispanic White	5,431	40.3 (38.6–42.0)	28.8 (27.3–30.4)	14.8 (13.7–16.0)	8.6 (7.8–9.4)	7.5 (6.6–8.5)
Non-Hispanic Black	2,287	35.9 (33.3–38.5)	29.8 (27.6–32.2)	16.5 (14.6–18.7)	8.8 (7.4–10.4)	9 (7.7–10.5)
Hispanic	2,315	43.6 (41.1–46.3)	31.0 (28.6–33.5)	13.2 (11.6–15.0)	6.6 (5.6–7.7)	5.6 (4.6–6.8)
Other	1,057	48.3 (44.1–52.4)	26.7 (23.5–30.1)	12.4 (10.4–14.7)	7.5 (5.6–10.0)	5.2 (3.7–7.1)
Education						
High school or less	6,141	39.3 (37.6–41.0)	29.2 (27.8–30.7)	14.6 (13.5–15.7)	9.0 (8.1–9.9)	7.9 (7.2–8.7)
Some college	3,554	41.8 (39.8–43.9)	29.8 (28.0–31.7)	14.8 (13.4–16.3)	6.8 (6.0–7.7)	6.8 (5.9–7.8)
4 years of college or more	1,395	48.3 (45.0–51.6)	28.4 (25.5–31.5)	13.5 (11.5–15.9)	6.1 (4.9–7.6)	3.6 (2.7–4.8)
Employment status						
Employed	4,889	48.9 (47.1–50.7)	32.0 (30.4–33.6)	11.8 (10.7–13.0)	5.1 (4.5–5.8)	2.3 (1.9–2.7)
Retired	374	21.8 (17.3–27.1)	27.4 (21.9–33.7)	17.8 (13.9–22.6)	17.6 (13.6–22.6)	15.4 (11.3–20.5)
Not working	5,827	34.8 (33.1–36.5)	27.0 (25.7–28.4)	16.9 (15.7–18.2)	10.2 (9.3–11.2)	11.1 (10.1–12.2)
Marital status						
Married/living with partner	3,888	40.9 (38.9–43.0)	30.0 (28.3–31.7)	14.7 (13.4–16.1)	7.5 (6.7–8.4)	7.3 (6.5–8.2)
Not married	7,202	41.0 (39.5–42.6)	28.9 (27.5–30.3)	14.4 (13.4–15.5)	8.4 (7.6–9.2)	6.9 (5.9–8.0)

2018–2022 National Health Interview Survey. Conditions Studied: Hypertension, hyperlipidemia, coronary heart disease, angina, heart attack, stroke, diabetes, and obesity. Participants: 11,090 adults (19–64 years) with Medicaid coverage.

**Figure 1:**
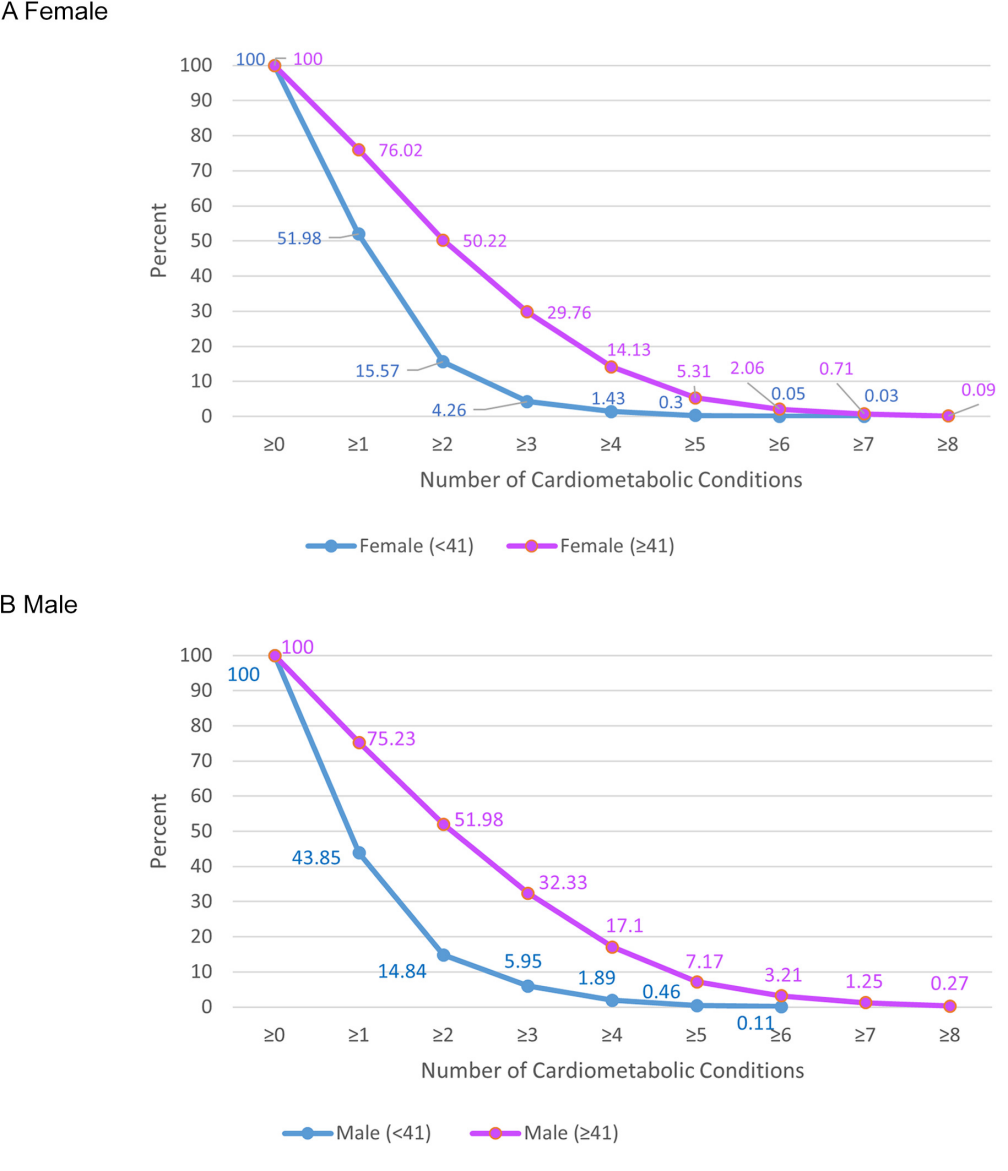
Summed proportions of Medicaid-insured adults with the indicated number of cardiometabolic conditions. 2018–2022 National Health Interview Survey. Conditions Studied: Hypertension, hyperlipidemia, coronary heart disease, angina, heart attack, stroke, diabetes, and obesity. Participants: 11,090 adults (19–64 years) with Medicaid coverage.

To explore differences in the distribution of cardiometabolic multimorbidity by sex and age category, Figure 1 depicts the unweighted CDF for the total number of diagnoses among Medicaid enrollees in the sample. Each level of total diagnoses along the horizontal axis is aligned with the proportion of enrollees with that level of diagnoses or greater (e.g., probability of ≥2 conditions) separately by sex and age category. While more women have 1 condition and more men have multimorbidity for adults both 19–40 years of age and 41–64 years of age, the differences in the proportions for older men and women are smaller in magnitude.

**Figure 2:**
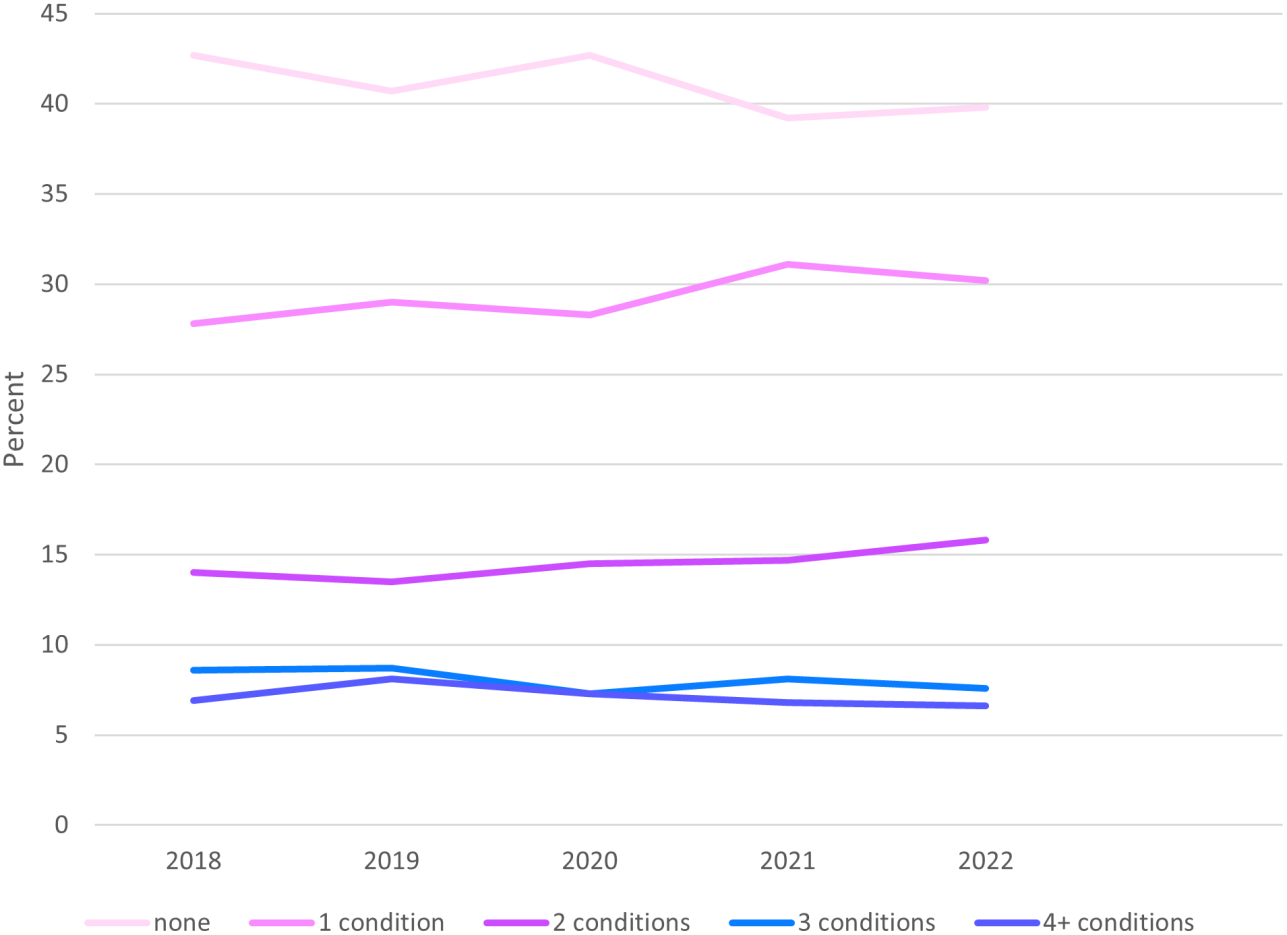
National trends in total cardiometabolic conditions among non-elderly adult Medicaid enrollees, 2018–2022. 2018–2022 National Health Interview Survey. Conditions Studied: Hypertension, hyperlipidemia, coronary heart disease, angina, heart attack, stroke, diabetes, and obesity. Participants: 11,090 adults (19–64 years) with Medicaid coverage.

Over the observed sample period, proportions of no conditions decreased while proportions of 1 condition increased (Figure 2). Proportions of 2, 3 and 4 or more diagnoses remained stable overall.

**Figure 3:**
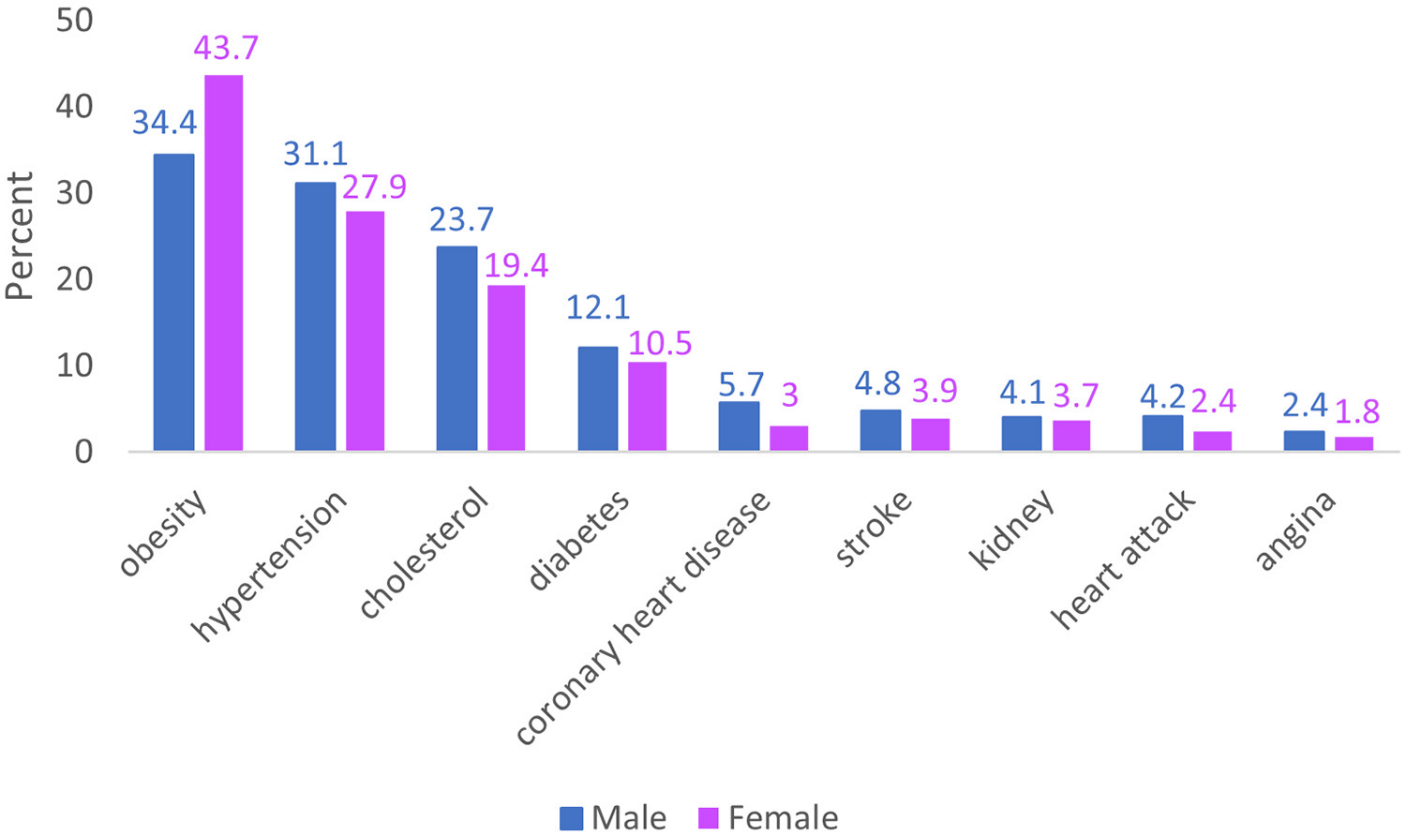
Prevalence of select cardiometabolic conditions in Medicaid-insured non-elderly adults in the US 2018–2022 by sex. 2018–2022 National Health Interview Survey. Conditions Studied: Hypertension, hyperlipidemia, coronary heart disease, angina, heart attack, stroke, diabetes, and obesity. Participants: 11,090 adults (19–64 years) with Medicaid coverage.

Turning to the prevalence of specific conditions, for both sexes obesity is the most common condition (34.4%–43.7%) followed by hypertension (27.9%–31.1%) and hyperlipidemia at (19.4%–23.7%), Figure 3. When considering both sex and age groups, younger and older women have higher rates of obesity alone (48.5% and 77.7% respectfully older and younger) than men (33% and 68.7% respectfully older and younger) (Table 2). The reverse is true for hypertension which ranges between 12.5%–26.3% for women versus 15.7%–32.9% cut for men. Similarly, hyperlipidemia ranges between 5.5%–19.7% for women versus 8.5%–24.8% for men.

Turning to specific conditions among adults with multimorbidity of 2 conditions (dyads), hypertension and obesity were the most common dyad for men and women but a relatively lower proportion for men. Instead, overall men were more likely to have hyperlipidemia and hypertension 22.3 compared to women (14.1%) which is the second most common dyad for both sexes. The most common combinations of conditions among adults with 2 and 3 conditions (dyads and triads) varied within sex by age group; for example, the top three dyads for women <41 were hypertension and obesity (55.1%), hyperlipidemia and obesity (17.2%), and diabetes and obesity (9.8%), whereas for women ≥41 the top 3 dyads were hypertension and obesity (37.4%), hyperlipidemia and hypertension (21.7%), and hyperlipidemia and obesity (13.2%).

**Table 2:** Top five cardiometabolic conditions and condition combinations among Medicaid non-elderly adult enrollees, 2018–2022.

Total conditions	Everyone				Female				Male			
	Cardiometabolic condition	Age		Total	Cardiometabolic condition	Age		Total	Cardiometabolic condition	Age		Total
		19–40	41–64			19–40	41–64			19–40	41–64	
Single	Obesity	74.8	42.6	63.1	Obesity	77.7	48.5	67.7	Obesity	68.7	33	54.2
	Hypertension	13.5	28.8	19.1	Hypertension	12.5	26.3	17.2	Hypertension	15.7	32.9	22.7
	Hyperlipidemia	6.4	21.7	12	Cholesterol	5.5	19.7	10.3	Cholesterol	8.5	24.8	15.1
	Diabetes	2.3	3.3	2.7	Diabetes	1.8	2.1	1.9	Diabetes	3.3	5.3	4.1
	Stroke	1.4	1.5	1.4	Stroke	1	1.2	1	Stroke	2.3	2.2	2.2
Dyad	Hypertension and Obesity	54.4	33.4	41.9	Hypertension and Obesity	55.1	37.4	45.1	Hypertension and Obesity	52.9	27.6	36.5
	Hyperlipidemia and Hypertension	6	24.7	17.1	Hyperlipidemia and Obesity	17.2	13.2	15	Hyperlipidemia and Hypertension	9.4	29.2	22.3
	Hyperlipidemia and Obesity	18	13.5	15.3	Hyperlipidemia and Hypertension	4.3	21.7	14.1	Hyperlipidemia and Obesity	19.7	13.9	15.9
	Diabetes and Obesity	8.3	5.7	6.7	Diabetes and Obesity	9.9	7.2	8.3	Diabetes and Hypertension	4.1	6.2	5.5
	Diabetes and Hypertension	2.8	5.7	4.5	Diabetes and Hypertension	2.2	5.3	4	Diabetes and Hyperlipidemia	2.5	5.5	4.4
Triad	Hyperlipidemia, Hypertension, Obesity	43	36	37.5	Hyperlipidemia, Hypertension, Obesity	44	39.9	40.7	Hyperlipidemia, Hypertension, Obesity	42	30.4	33.1
	Diabetes, Hypertension, Obesity	27.7	11.6	15	Diabetes, Hypertension, Obesity	25.9	12.5	15.1	Diabetes, Hyperlipidemia, Hypertension	6.3	17.5	14.9
	Diabetes, Hyperlipidemia, Hypertension	4.8	16.6	14.1	Diabetes, Hyperlipidemia, Hypertension	3.5	16	13.5	Diabetes, Hypertension, Obesity	29.7	10.3	14.9
	Diabetes, Hyperlipidemia, Obesity	6.8	8.4	8.1	Diabetes, Hyperlipidemia, Obesity	5.7	9	8.4	Diabetes, Hyperlipidemia, Obesity	8.1	7.5	7.6
	Hyperlipidemia, Hypertension, Stroke	0	4.6	3.6	Hypertension, Stroke, Obesity	7.9	3.6	4.4	Hyperlipidemia, Hypertension, Stroke	-	7.1	5.4

2018–2022 National Health Interview Survey. Conditions Studied: Hypertension, hyperlipidemia, coronary heart disease, angina, heart attack, stroke, diabetes, and obesity. Participants: 11,090 adults (19–64 years) with Medicaid coverage.

Dyad is multimorbidity of 2 conditions and triad is multimorbidity of 3 conditions.

## Discussion

We find that 29.6% of Medicaid-insured non-elderly adults experience cardiometabolic multimorbidity of various levels during 2018–2022. Obesity, hypertension and hyperlipidemia dominate Medicaid-insured individuals with one or more cardiometabolic conditions. In previous research that explored obesity-related multimorbidity (coronary heart disease, hypertension, stroke, diabetes) in the broader US population 2007–2016, researchers reported that 12.3% of the population ages 40–79 had multimorbidity and that the most common conditions were hypertension and diabetes (11). Excluding obesity and hyperlipidemia, we also find that hypertension and diabetes were the most prevalent despite using a younger sample (19–64).

We found that rates of cardiometabolic multimorbidity at 2, 3, and 4 or more conditions have remained constant over the observed period, but that rates for one condition increased while rates for no conditions decreased. The increase could reflect increased diagnosis rates from improved access to care in the post-ACA US healthcare system or increasing trends in cardiometabolic conditions. We note that increases in obesity in our data would not indicate more diagnoses and access to healthcare because obesity is constructed from self reported height and weight information.

Further, proportions of any cardiometabolic multimorbidity and severity of cardiometabolic multimorbidity (total conditions) reflected disparities in social determinants of health (SDOH) despite the common source of health insurance. Along with previous studies documenting within program disparities in cardiometabolic disease control and increased risk for more severe cardiometabolic morbidity, the disparities highlight the need for integrated SDOH interventions in Medicaid programs (12–14). Addressing SDOH was a major priority for state Medicaid programs before the start of the second Trump Administration (15).

Our findings also show that the clinical needs of men and women insured by Medicaid differ, especially among younger adults. Medicaid-insured women are more likely to have a single condition and were more likely to have obesity relative to Medicaid-insured men who were more likely to have multimorbidity and all other cardiometabolic conditions included in the study. The difference between sexes was larger and more consistent for younger adults (<41 years). The prevalence of obesity highlights an opportunity for preventive interventions in Medicaid programs using new weight-loss drugs (16). Proposed changes to Medicaid funding may instead cut federal Medicaid spending, potentially forcing states to retract Medicaid eligibility expansions (17).

Addressing the unique needs of patients with multimorbidity requires system reform. There are opportunities to improve care and reduce costs from avoidable complications that result from interactions between conditions and their treatments, as well as opportunities to reduce costs from lower rates of condition-related functional impairment (18). Addressing care for Medicaid-insured patients with multimorbidity may also bolster broader reforms as Medicaid is the single largest insurer in the country-although the kind of care received by Medicaid enrollees’ with multimorbidity can vary across state programs.

## Limitations

In 2020, NHIS survey efforts were adversely affected by the onset of the COVID-19 pandemic, which could affect our estimates. Additionally, NHIS data do not allow us to detect undiagnosed cardiometabolic conditions, potentially underestimating true prevalence. Because NHIS excludes individuals with no fixed household address (e.g., homeless and/or transient persons not residing in shelters), the sample limits representation of the Medicaid population (10). Finally, since all comorbidity data in the study were obtained through a questionnaire, there is the possibility of response bias. Future work using the restricted NHIS state identifiers will allow for analysis of the widely documented heterogeneity in state Medicaid program design, eligibility and generosity (19, 20). Currently, most states have expanded Medicaid eligibility under the ACA. Non-expansion states predominantly account for much of the Southeastern US and would not have non-elderly, childless adults without disability in our national sample.

## Conclusions

A stable 30% of Medicaid-insured non-elderly adults have cardiometabolic multimorbidity dominated by obesity, hypertension and hyperlipidemia. Differences in the number of conditions and types of conditions across subpopulations offer opportunities for targeted interventions to prevent and control cardiometabolic multimorbidity. Instead, proposed cuts to federal Medicaid spending may exacerbate the burden of cardiometabolic multimorbidity in low-income populations.

## Data Availability

Publicly available datasets were analyzed in this study. This data can be found here: https://www.cdc.gov/nchs/nhis/index.html.
